# Connexin Expression in Human Minor Salivary Glands: An Immunohistochemical Microscopy Study

**DOI:** 10.3390/molecules27185926

**Published:** 2022-09-12

**Authors:** Alessandra Falleni, Stefania Moscato, Giovanni Fulvio, Enza Polizzi, Margherita Bernardeschi, Francesco Bianchi, Valentina Donati, Manuela Cabiati, Chiara Ippolito, Silvia Del Ry, Chiara Baldini, Letizia Mattii

**Affiliations:** 1 Department of Clinical and Experimental Medicine, University of Pisa, 56126 Pisa, Italy; 2 Pathological Anatomy Unit, Azienda Ospedaliero-Universitaria Pisana, 56126 Pisa, Italy; 3 Institute of Clinical Physiology (IFC), National Research Council (CNR), 56124 Pisa, Italy

**Keywords:** connexin 26, connexin 32, connexin 43, immunofluorescence, immunoelectron microscopy, human salivary glands, RT-PCR, myoepithelial cell

## Abstract

Connexins (Cxs) are transmembrane proteins involved in the formation of hemichannels and gap junctions (GJs). GJs are involved in various physiological functions, including secretion in glandular tissue. It has been demonstrated that Cx26, Cx32, and Cx43 are mainly expressed in glands, but no data are available in human salivary glands to date. The aim of our study was to investigate the presence and the localization of Cxs in human minor labial salivary glands. Immunofluorescence and immunoelectron microscopy were employed to evaluate the Cx26, Cx32, and Cx43 protein in human labial salivary gland biopsies (hLSGBs). RT-PCR was also used to detect their mRNA expression. Cx expression was found at both the mRNA and protein levels in all hLSGBs analysed. Cxs were observed at the level of the duct and acinar cells, as well as in myoepithelial cells. The localization of the three Cx types was very similar, suggesting colocalization of these Cxs in the same connexons. These results demonstrated the presence of Cxs in human salivary glands for the first time. Moreover, the few samples with primary Sjögren’s Syndrome analysed only by immunofluorescence showed an alteration of the Cx expression, indicating that these proteins could be involved in salivary gland dysfunctions.

## 1. Introduction

Connexins (Cxs) are membrane-spanning proteins that form single transmembrane channels called connexons or hemichannels. When a connexon docks with a connexon from an opposing cell, it forms an intercellular channel that links the cytoplasm of the two neighboring cells. Plaques containing several intercellular channels spanning the two plasma membranes represent gap junctions (GJs). Each connexon that is composed of six Cxs can be homomeric if the Cxs are of a single type or heteromeric if the Cxs are of multiple types. As a result, an intercellular channel can be both homotypic, formed of two identical homomeric connexons, and heterotypic, formed of two different homomeric connexons or of two heteromeric connexons. At least 21 isoforms of Cxs are expressed in humans, and they are found in almost every tissue, generally more than one Cx type per tissue. The role of hemichannels is to exchange ions and small molecules between the cytosol and the extracellular space, while GJs are responsible for the direct diffusion of these molecules between adjacent cells. Channels composed of different Cx isoforms show specific and characteristic molecular permeability [1,2,3]. Therefore, these channels are involved in various physiological functions, including glandular secretion [4,5,6,7,8]. In most glands, Cx26, Cx32, and Cx43 are the ones mainly expressed. However, with regard to salivary glands, there are limited investigations on the expression of Cxs. Such studies demonstrated the expression of Cx26, Cx32, and Cx43 at the level of rat and mouse major salivary glands [7,9], as well as the Cx32 and Cx43 involvement in developing rat submandibular salivary glands [10]. To date, no study has been performed on the expression of Cxs in human salivary glands, apart from our previous preliminary study demonstrating the modulation of the Cx26 expression in human minor salivary glands from Sjögren’s Syndrome (SS) patients [11]. The aim of this study was to further investigate the presence and localization of Cx26, Cx32, and Cx43 in normal human labial salivary glands (hLSGs) with the ultimate objective of recognizing a possible Cx aberrant subcellular localization in hLSGs from patients with salivary gland diseases, and to assess the Cx role in salivary gland dysfunction and hyposalivation. The identification and characterization of the Cx expression in minor salivary glands are important because, being that the biopsies of these glands are used for diagnosing diseases, such as SS and cystic fibrosis [12], the same biopsies may be used to study and monitor Cx expression in these diseases. LSGs are types of minor salivary glands mainly localized beneath the mucosal epithelium of the lips. The hLSGs generally consist of one duct that houses serous and mucous acini, which continuously secrete mostly mucous and serous substances to maintain oral health [12]. For our study, we used hLSG biopsies (hLSGBs) that were obtained as part of the routine diagnostic procedures when primary Sjögren’s Syndrome (pSS) was suspected. Samples without pSS (NS) and with mild or absent non-focal sialoadenitis were mainly selected for the study. Also, a few samples from pSS patients (SS) were tested for Cx detection. Immunofluorescence (IF) and immunoelectron (IE) microscopy were employed to evaluate Cx protein expression, while RT-PCR was used to detect their mRNA expression.

## 2. Results and Discussion

Due to the lack of literature data about Cx expression in human salivary glands, we aimed to investigate the presence and the distribution of Cxs in human gland tissue, starting from Cx26, Cx32, and Cx43, which were found expressed in the major salivary glands of rats [7,9,10]. We mostly used NS-hLSGBs, which were obtained from patients in whom the diagnosis of pSS was ruled out. A few samples from the pSS samples were also investigated.

Immunohistochemical analyses were performed in agreement with the relevant reports on Cx immunohistochemistry [13,14]. The results of this paper were obtained from at least three repeated experiments, and the evaluation was conducted by two blinded, independent researchers. The specific Cx rabbit primary antibodies employed for immunohistochemical analyses were yet previously tested and used by our research group on cardiac and liver tissues [15,16], which, therefore, represent positive control tissues for our primary antibodies. Moreover, as demonstrated by the results obtained on the negative controls carried out in the present work (see Section 3.2.1 and Section 3.2.2), the specificity of all used antibodies was confirmed. Indeed, the irrelevant primary antibody was demonstrated not to label Cxs, and the two secondary antibodies were confirmed to be specific to their individual primary antibodies (Figure S1).

### 2.1. Cx Protein IF Analysis

**Figure 1:**
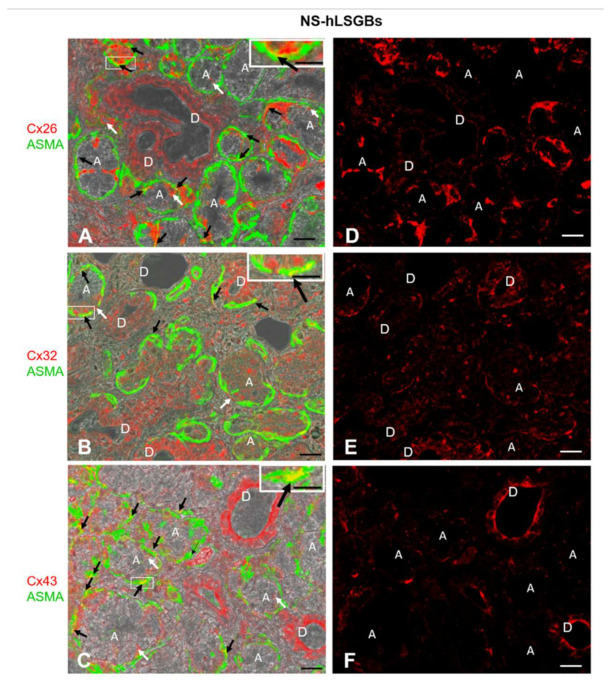
IF analysis for Cx26, Cx32, and Cx43 in NS-hLSGBs. (**A**–**C**) Representative confocal microscopy images of double IF with transmitted light contrast. Merged images show Cx positivity in red, ASMA positivity in green, and Cx-ASMA colocalization in yellow. These images have been modified by enhancing the saturation in order to better appreciate the yellow stain. White arrows indicate red immunoreaction at the plasma membrane of the duct cells and at the basal/basolateral side of the acinar cells. Black arrows point to the yellow spot due to the colocalization of Cx and ASMA. Inserts show the higher magnification of the squares. (**D**–**F**) Single IF images representing Cx immunoreactions (red) corresponding to (**A**–**C**) microscopic fields. A, acinus; D, duct. Scale bars, 20 µm; Inserts, 10 µm.

The results obtained by immunofluorescence demonstrated that all three Cxs were present at the level of all analyzed six NS-hLSGBs. Interestingly, the different Cx isotypes showed a similar cell localization, though with few differences (Figure 1).

Cx immunoreactivity was observed at the level of the plasma membrane of the duct cells, as well as at the basal and basolateral side of the acinar cells (Figure 1A–C, the white arrows). In particular, Cx32 and Cx43 immunofluorescence at the duct cells seemed higher than that observed for Cx26, which, conversely, appeared more expressed at the basal side of the acinar cells (Figure 1D–F). However, the quantification of Cx relative expression levels was not considered suitable, bearing in mind the application methods of this IF and the aim of our qualitative work. For example, the differences in antibody affinities and epitope accessibilities potentially contribute to the relative Cx immunoreactivity amounts. The qualitative results obtained in this study will enable the research of quantitative differences in the expression of the same Cx among different samples, i.e., normal and pathological samples. By these IF reactions, it has not been possible to distinguish serous from mucous acini. However, as mentioned below, IE microscopy allowed us to demonstrate the presence of Cxs in both types of acini. Cx immunoreactivity was also found in myoepithelial cells. Myoepithelial cells (Figure 1 in green) have been identified by their immunoreactivity to the antibody anti-Alpha Smooth Muscle Actin (ASMA), and the colocalization of ASMA and Cx demonstrated the presence of Cxs in these cells (Figure 1A–C, yellow staining pointed to by black arrows). These star-shaped cells, lying between the basal lamina and the acinar or ductal cells, are involved in various functions, including: (1) the salivary gland differentiation during embryonic development by secreting growth factors, (2) the facilitation of gland secretion by means of their contraction, (3) the maintenance of gland patency, which may be lost during masticatory movements, (4) the transportation of metabolites in the secretory process, and (5) the formation and maintenance of the basement membrane [17]. Therefore, Cxs in myoepithelial cells could be involved in some of these functions. In this regard, in mammary glands, it has been demonstrated that a specific asymmetric expression of Cxs between luminal epithelial and myoepithelial cells is essential for the optimal contractile function of the mammary gland [18].

**Figure 2:**
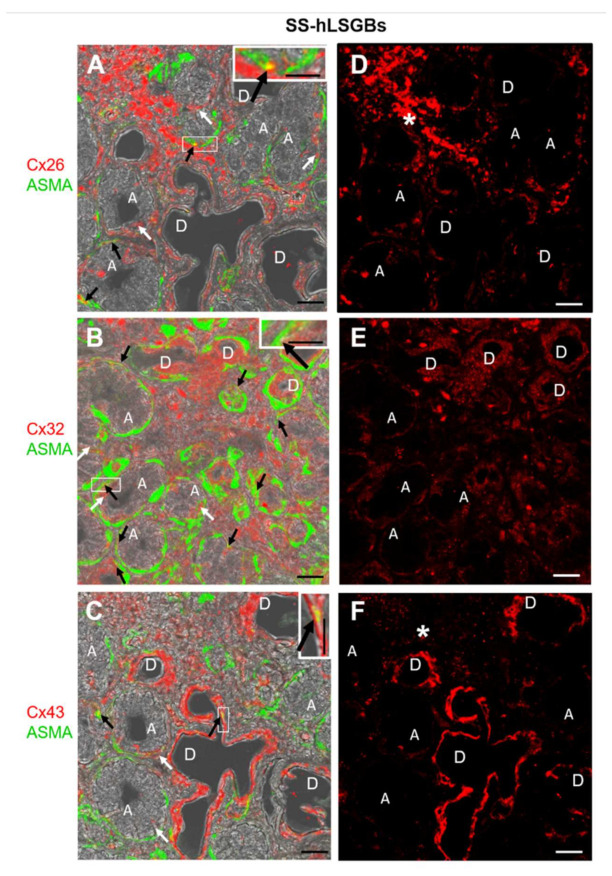
IF analysis for Cx26, Cx32, and Cx43 in SS-hLSGBs. (**A**–**C**) Representative confocal microscopy images of double IF with transmitted light contrast. Merged images show Cx positivity in red, ASMA positivity in green, and Cx-ASMA colocalization in yellow. These images have been modified by enhancing the saturation in order to better appreciate the yellow stain. White arrows indicate red immunoreaction at the plasma membrane of the duct cells and at the basal/basolateral side of the acinar cells. Black arrows point to the yellow spot due to the colocalization of Cx and ASMA. Inserts show the higher magnification of the squares. (**D**–**F**) Single IF images representing Cx immunoreactions (red) corresponding to (**A**–**C**) microscopic fields. Asterisks (*) point to the inflammatory cells. A, acinus; D, duct. Scale bars, 20 µm.

Cx immunofluorescence analysis was also performed on four samples from patients with pSS (SS-hLSGBs). The results showed that the Cx localization was similar to that of NS-hLSGBs but with an apparent different level of expression depending on the Cx type (Figure 2). Indeed, at least by the optical microscopical observation, Cx26 immunoreactivity appeared reduced in SS-hLSGBs (Figure 2D) compared to NS-hLSGBs (Figure 1D) at the acinar level, while Cx32 and Cx43 immunofluorescences seemed to increase in SS-hLSGBs (Figure 2E,F) rather than in NS-hLSGBs (Figure 1E,F) at the level of the duct cells. Inflammatory cells observed in SS-hLSGBs showed immunopositivity for Cx26 and Cx43 (Figure 2D,F asterisks).

These results are interesting since they could suggest the involvement of Cxs in the pSS and confirm our previous results on Cx26 [11]. However, before discussing the possible pathogenetic role of the Cxs/GJs in pSS-salivary gland dysfunction, further studies are necessary to better corroborate these preliminary results. In particular, we first need to analyze a larger number of NS-hLSGBs and SS-hLSGBs samples and use the image analysis for the semi-quantitative evaluation of the immunoreactivity.

### 2.2. Cx Protein IE Microscopy

**Figure 3:**
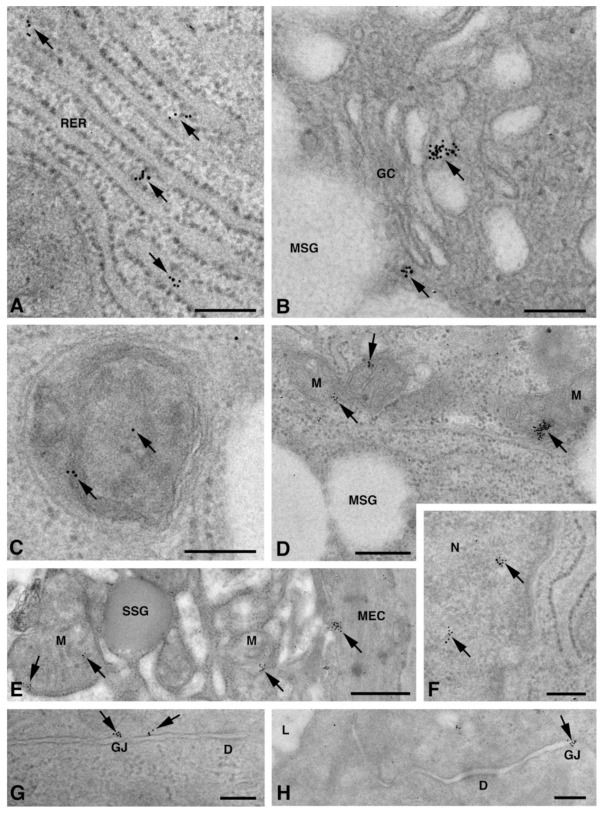
TEM immunogold analysis for Cx26 in NS-hLSGB. Representative images of immunoreactions (arrows) at the level of different cell compartments: rough endoplasmic reticulum (**A**), Golgi complex (**B**), and autophagic vacuole (**C**) in mucous cells; mitochondrial inner membrane and cristae in mucous (**D**) and serous (**E**) cells; nucleus (**F**) and apparent GJ in the lateral plasma membranes between the mucous cells (**G**). In (**E**), gold particles are also visible on the plasma membrane of a myoepitelial cell (MEC) bordering the serous cell. In (**H**), immunogold reaction is on an apparent GJ between the lateral plasma membranes of two duct cells. D, desmosome; GC, Golgi complex; L, lumen; M, mitochondrium; MEC, myoepithelial cell; MSG, mucous secretory granule; N, nucleus; RER, rough endoplasmic reticulum; SSG, serous secretory granule. Scale bars (**A**–**D**) and (**F**–**H**), 200 nm; (**E**) 500 nm.

**Figure 4:**
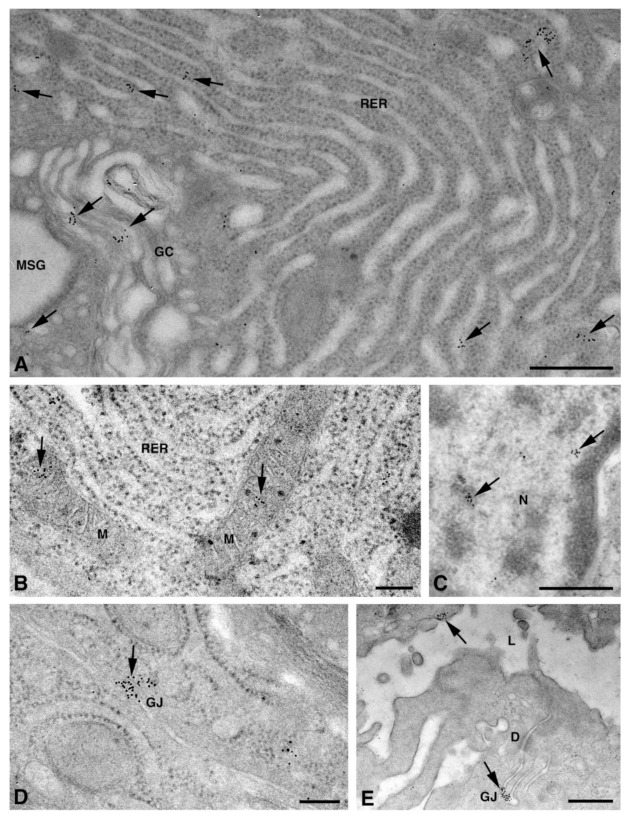
TEM immunogold analysis for Cx32 in NS-hLSGB. Representative images of immunoreactions (arrows) at the level of different cell compartments: rough endoplasmic reticulum and Golgi complex (**A**), mitochondria (**B**), nucleus (**C**), and apparent GJ in the lateral plasma membranes (**D**) of mucous cells. In (**E**) gold particles are visible on the apical luminal membrane of a duct cell and on a GJ between the lateral plasma membranes of two duct cells. D, desmosome; GC, Golgi complex; L, lumen; M, mitochondrium; MSG, mucous secretory granule; N, nucleus; RER, rough endoplasmic reticulum. Scale bars (**A**,**C**,**E**), 500 nm; (**B**) and (**D**), 200 nm.

**Figure 5:**
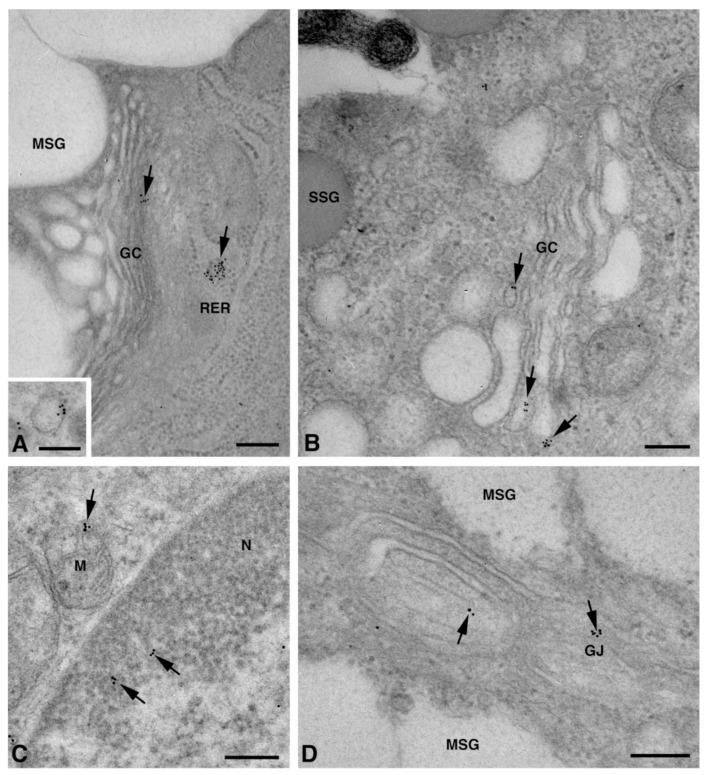
TEM immunogold analysis for Cx43 in NS-hLSGB. Representative images of immunoreactions (arrows) at the level of different cell compartments: rough endoplasmic reticulum, Golgi complex, and a Golgi-derived vesicle (insert) in a mucous cell (**A**), Golgi complex in a serous cell (**B**), mitochondria and nucleus in a serous cells (**C**), and GJ in the lateral plasma membranes of mucous cells (**D**). GC, Golgi complex; L, lumen; M, mitochondrium; MSG, mucous secretory granule; N, nucleus; RER, rough endoplasmic reticulum; SSG, serous secretory granule. Scale bars (**A**–**D**), 200 nm; (**A**) insert, 170 nm.

One of the NS-hLSGBs observed in IF analysis has also been examined by transmission electron microscopy (TEM). The use of a different immunohistochemical method to identify Cxs, and specifically the IE analysis, allowed us to confirm the results obtained by IF. Moreover, TEM observation enabled us to better clarify the cellular and subcellular localization of the tested Cxs. In the NS-hLSGB, the three Cxs were observed at the level of the serous and mucous acinar cells, as well as at the duct cell level. Immunogold particles were found in the endoplasmic reticulum, Golgi complex, Golgi-derived vesicles, and autophagosomes that were localized in the basal side of the cells (Figure 3A–C, Figure 4A and Figure 5A,B). These findings were expected since, in these organelles, Cxs are in sequence, synthesized, oligomerized into hexamers, included in the vesicles, translocated to the cell membrane or to other cell compartments, and then degraded [19]. In addition, Cxs were also found in non-canonical cell structures, including the mitochondria and nuclei (Figure 3D–F, Figure 4B,C and Figure 5C) according to that previously found in other tissues with regard to Cx43 and Cx26 [20,21,22]. The nuclear Cx functions are still unknown, but their involvement in the regulation of transcription and, consequently, in cell growth and differentiation has been suggested for Cx43 and Cx26 [20,23]. Regarding mitochondria Cx functions, it has been demonstrated that cardiomyocyte Cx43 forms hemichannels, which may regulate potassium uptake, mitochondrial uncoupling, and ROS production [24]. The expression of Cx26, Cx32, and Cx43 at the LSG level has been proved for the first time in this study. Even though the nuclear and mitochondrial function of LSG-Cx26, -Cx32, and -Cx43 is unknown, we cannot exclude that they play similar roles to the ones described above. Cx immunolabeling was also observed in the plasma membrane. In acinar serous and mucous cells, Cxs were observed at the basal and basolateral membrane (Figure 3G, Figure 4D and Figure 5D). In the lateral membrane, mainly near the desmosomes, the Cx immunopositivity was observed at the GJ level between two adjacent cells. In the duct cells, Cx expression was found at the level of the lateral plasma membrane (Figure 3H and Figure 4E), but Cx32 was also detected at the luminal membrane (Figure 4E). While the role of Cxs in human salivary glands is still unknown, the distribution of Cxs at the membranes between the acinar cells of LSGs could suggest the involvement of Cx-made GJs in the regulation of the secretory function. Namely, GJs could allow the spread of excitation among the acinar cells within the gland. This function has been previously assumed for rat major salivary glands and mouse lacrimal glands [6,7].

**Figure 6:**
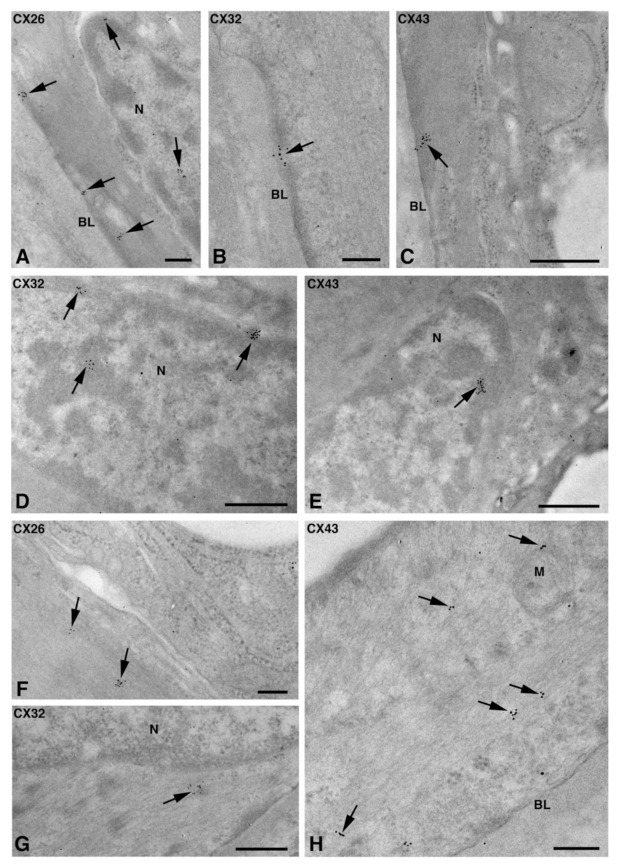
TEM immunogold analysis for Cx26 (**A**,**F**), Cx32 (**B**,**D**,**G**), and Cx43 (**C**,**E**,**H**) in NS-hLSGB myoepithelial cells. Immunoreactions (arrows) are visible at the level of the nucleus (**A**,**D**,**E**), the basal plasma membrane (**A**–**C**), among fibrils (**F**–**H**), and on the mitochondria (**H**). BL, basal lamina; M, mitochondrion; N, nucleus. Scale bars (**A**,**B**,**F**,**H**), 200 nm; (**C**–**E**,**G**) 500 nm.

In myoepithelial cells, the Cx immunolabeling was observed at the plasma membrane bordering the basal lamina (Figure 6A–C) and, occasionally, the acinar cells (Figure 3E and Figure 6D). Furthermore, the Cx immunoreaction was also found at the level of non-canonical structures, such as the nucleus (Figure 6A,D,E), cytoplasm filaments (Figure 6F–H), and mitochondria (Figure 6H). The presence of specific Cxs between the myoepithelial cells and acinar cells of the LSGs could be required for an appropriate contraction of myoepithelium, as has been demonstrated for mouse mammary glands [18]. The localization of the three types of Cxs at the level of the same subcellular structures seems to suggest the presence of heteromeric connexons or heterotipic intercellular channels. To confirm this hypothesis, double/triple immunohistochemical reactions and/or a proximity ligation assay should be carried out in future studies.

### 2.3. Cx mRNA Expression Analysis

**Figure 7:**
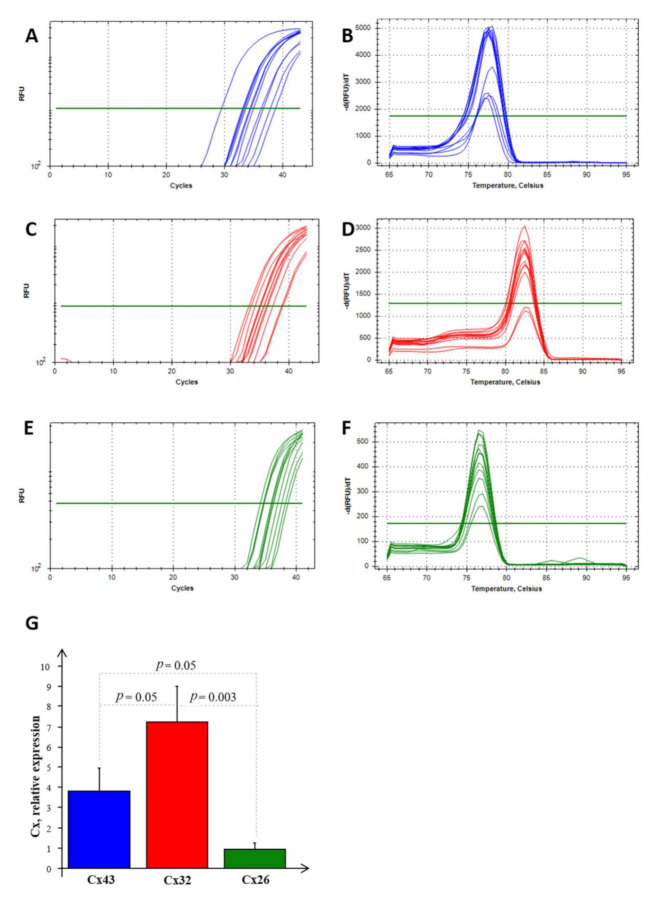
RT-PCR analysis for Cx26, Cx32, and Cx43 in NS-hLSGBs. Example of the threshold cycle (Ct), relative amplification curves, and Melting peak (the negative first derivative of the change in the fluorescence plotted as a function of temperature) for: Cx43 (blue) (**A**,**B**), Cx32 (red) (**C**,**D**), and Cx26 (green) (**E**,**F**); relative expression of Cxs in NS-hLSGB; data are expressed as means ± SEM (**G**).

Real-time PCR experiments allowed us to highlight the mRNA expression of Cx26, Cx32, and Cx43 in NS-hLSGBs, as reported in Figure 7. Normalizing the data with the three most stable reference genes (ACTB, RPL13a, and RPS4X, with an M value < 1) in NS-hLSGBs, the trend of the expression is different for each Cx analyzed but statistically significant only between Cx32 and Cx26 (Figure 7G).

These results confirmed that the Cx expression observed by means of IF analysis and IE microscopy is due to the synthesis of the relative Cx-mRNA in hLSGBs.

## 3. Materials and Methods

### 3.1. Salivary Gland Collection

hLSGBs were recruited at the Rheumatology Clinic of the University of Pisa as part of the routine diagnostic procedures when primary Sjögren’s Syndrome (pSS) was suspected. We selected seventeen hLSGBs that were obtained from thirteen patients (eleven women and two men, aged 45–72 years) without pSS (NS) and with a preserved unstimulated salivary flow rate (mean 0.467 ± 0.299 mL/min). Four hLSGBs from patients (women aged 57–62 years) with the diagnosis of pSS (SS), fulfilling the ACR/EULAR 2016 criteria [25], were also recruited. These SS patients complained of dry eyes and dry mouth and presented reduced unstimulated salivary flow (mean 0.299 ± 0.225 mL/min) and pathological ocular test results. At the time of saliva sampling, no patients or controls had a diagnosis of oral periodontitis. Moreover, histological evaluations of the LSG were performed on the Haematoxylin and Eosin (H&E) stained tissue sections, and the focus score ranged from 1.3 to 3.2. The study was approved by the Medical Research Ethics Committee of the University of Pisa (number 3060 and number 65,394). All the patients provided an informed consensus to participate in the study.

### 3.2. Immunohistochemical Analyses

#### 3.2.1. Immunofluorescence (IF) Microscopy

Ten hLSGBs from NS (six) and SS (four) patients were fixed in a neutral buffered formalin overnight at 4 °C and, after washing in PBS, they were paraffin-embedded. H&E staining and immunohistochemical reactions were performed on 5 µm thick sections. The sections were de-paraffined by xylene, rehydrated in absolute ethanol, washed in double distilled water (dd-H_2_O), and finally rinsed in PBS.

For IF analysis, antigen retrieval was performed by treating the tissue sections for 10 min with 0.2% triton-X100/PBS. Then, after 1 h in a blocking solution (BS, 0.1% Tween, and 0.25% BSA in PBS), samples were incubated overnight at 4 °C with a BS solution containing two primary antibodies, a rabbit anti-Cx antibody and the mouse anti-ASMA antibody. Primary antibodies were as follows: rabbit anti-human Cx26 antibody (1:100, RαCx26, NBP2-41304, Novus Biologicals, Abingdon, UK), rabbit anti-human Cx32 antibody (1:25, RαCx32, Invitrogen 71-0600, Waltham, MA, USA), rabbit anti-human Cx43 antibody (1:100, RαCx43, NB1000-91717, Novus Biologicals, Abingdon, UK), and mouse anti-human ASMA (1:400, A2547, Sigma Aldrich, Saint Louis, MO, USA). The slides were then washed three times in BS and incubated for 90 min in the dark with fluorescent anti-rabbit secondary antibodies diluted by 1:250 in BS (Alexa Fluor^®^ 568 donkey anti-rabbit and Alexa Fluor^®^ 488 goat anti-mouse, Invitrogen, Waltham, MA, USA). The samples were mounted with a dapi mounting medium (Invitrogen, Waltham, MA, USA). The entire procedure was performed at room temperature (RT) unless specified. The negative controls for the specificity of the secondary antibodies were performed by (1) omitting primary antibodies that were replaced by the BS solution and (2) using each primary antibody with matching and non-matching secondary antibodies. The negative control for the specificity of the primary antibodies was obtained using an irrelevant primary antibody (mouse anti-skeletal myosin antibody, 1:750, MαMyo M4276, Sigma-Aldrich, Saint Louis, MO, USA). Immunofluorescent reactions were observed by a confocal laser scanning microscope at 40x magnification (TC SP8 Leica Microsystems, Mannheim, Germany) and images (at least 30 for each sample) were obtained using a sequential scan mode with 488 nm and 568 nm laser lines and transmission light.

#### 3.2.2. Immunoelectron (IE) Microscopy Post-Embedding Technique

One NS-hLSGB sample was fixed in 1% (*w*/*v*) glutaraldehyde-4% (*w*/*v*) paraformaldehyde in a phosphate-buffered saline (PBS 0.1 M and pH 7.4) for 2 h at 4 °C, and, after washing it in the same buffer, the specimen was postfixed in 1% (*w*/*v*) OsO_4_/PBS for 1 h at RT. This method, which combines aldehyde and mild OsO_4_, allows for a minimal cover of antigen epitopes while preserving the cell architecture and sub-cellular structures [21]. NS-hLSGB was then washed in PBS, dehydrated in a graded series of ethanol, and transferred to a propylene oxide for 6 min. Finally, the sample was embedded in epon-araldite, in a flat mold at 60 °C for 72 h. Ultrathin sections (60–80 nm) were obtained with a Reichert-Jung Ultracut E equipped with a diamond knife and collected on 200-mesh formvar/carbon-coated nicKel grids. The grids were incubated with a NaIO_4_ saturated aqueous solution for 30 min at RT to partially remove the OsO_4_ and unmask the antigens [26]. To block non-specific antigenic sites, nickel grids were incubated in a cold PBS-blocking solution containing 10% of normal goat serum (NGS) and 0.2% saponin for 20 min. The grids were then incubated overnight in a humidified chamber at 4°C with a single primary antibody, RαCx26 or RαCx32 or RαCx43 (diluted at 1:50 in 1% NGS/0.2% saponin/PBS). After rinsing in cold PBS, the grids were incubated with the 10 nm gold-conjugated anti-rabbit antibody (diluted at 1:20 in 1% NGS/0.2% saponin/PBS; AC-10-01-05, Cytodiagnostics, Burlington, ON, Canada) for 1 h at RT. After washing in PBS, ultrathin sections were treated with 1% glutaraldehyde for 3 min, washed in distilled water to remove salt traces, and counterstained with uranyl acetate and lead citrate. The negative controls for the specificity of the secondary antibody were performed by (1) omitting the primary antibody and incubating the sections with the secondary antibody and (2) using a non-matching secondary antibody (10 nm gold-conjugate goat anti-mouse antibody at 1:20 in 1% NGS/0.2% saponin/PBS; ab27242, Abcam, Cambridge, UK). The negative control for the primary antibodies was performed using an irrelevant primary antibody (mouse anti-skeletal myosin antibody, 1:50, MαMyo, M4276, Sigma-Aldrich, Saint Louis, MO, USA). Ultrathin sections were analyzed using a Jeol 100SX (Japan) transmission electron microscope operating at 80 KV. Micrographs were obtained with an AMTXR80b Camera System. At least 50 images were analyzed for each of the samples.

### 3.3. RNA Extraction and Real Time PCR Analysis

Glandular tissues from seven NS samples were homogenized with an automated tissue lyser (TissueLyser Mixer Mill MM300, Qiagen S.p.A, Milano, Italy) and were homogenized with a guanidinium thiocyanate–phenol solution (Qiazol, Qiagen SpA, Milano, Italy) following the miRNeasy Mini kit manufacturer’s instruction (Qiagen SpA, Milano, Italy). High-quality RNA was then eluted in 40 µL of RNAse-free water, as previously described [27,28]. All RNA samples were stored at −80 °C after the integrity, purity, and concentration evaluation. cDNA was reverse transcribed with an iScript™ cDNA Synthesis kit (Bio-Rad Laboratories Inc., Hercules, CA, USA), according to the manufacturer’s instructions, starting from 1 μg of total RNA extracted from salivary glands, as a template. The reverse transcription reaction consisted of incubation at 25 °C for 5 min, followed by three different cycles at 4 °C for 30 min and 45–48 °C for 10 min. The reverse transcriptase enzyme was inactivated by heating to 85 °C for 5 min. The cDNA samples obtained were stored at 4 °C until further use. Real-time PCR reactions were performed in duplicate in the Bio-Rad C1000™ thermal cycler (CFX-96 Real-Time PCR detection systems, Bio-Rad Laboratories Inc., Hercules, CA, USA), as previously described [15]. The melting curves were generated from 65 °C to 95 °C with increments of 0.5 °C/cycle to assess product specificity. The primer sequences for the reference genes, Cx32 and Cx43, were designed through the use of a dedicated software, Beacon Designer^®^ (version 8.1, Premier Biosoft International, Palo Alto, CA, USA) able to compare the nucleotide sequences contained in the GenBank database of the NCBI (http://www.ncbi.nlm.nih.gov/Genbank/index.html) and synthesized by the Sigma Aldrich company (Merk group, D), as reported in Table 1. Whenever possible, intron-spanning primers were selected to avoid the amplification of genomic DNA. Given the difficulty of designing specific oligonucleotides for Cx26, and to avoid confounding effects due to the poor ability to discriminate an appropriate coding region, they were developed in collaboration with a specialized company (QIAGEN, SpA, Hilden, Germany). Details of the pre-cust primers are given in Table 1.

#### Statistical Analysis

The GeNorm technology integrated into Bio-Rad’s CFX96 manager software (CFX-96 Real-Time PCR detection systems, Bio-Rad Laboratories Inc., Hercules, CA, USA) was used to establish the most stably expressed genes, as described by Vandesompele et al. [29].

The statistical analysis of the results was carried out through the Stat-View 5.0.1 software released for Windows Statistical (1992-98, SAS Institute Inc., SAS Campus Drive, Cary, NC, USA). The mRNA expression data were normalized by the geometric mean of the three most stably expressed genes (ACTB, RPL13a, and RPS4X), and the relative quantification was performed by the ΔΔCt method. A Fisher’s test, after ANOVA, was used to obtain the results that were reported in the bar chart and expressed as mean ± SEM; a *p* < 0.05 was considered significant.

When mRNA values were not normally distributed, the statistical analysis was conducted after the logarithmic transformation of the data.

## 4. Conclusions

Knowledge of Cx expression in salivary glands is currently restricted to the rodent major salivary glands [7,9,10]. In the present study, we demonstrated for the first time the mRNA and protein expression of Cx26, Cx32, and Cx43 in human minor salivary glands, also exploring their cellular and subcellular localization. These findings suggest that Cx expression in human minor salivary glands may be compared to that found in rodent salivary glands. Therefore, as for rodents, a Cx involvement in salivary gland development and secretory function can be suggested, as well as the presence of heteromeric connexons. The latter is supported by the already found colocalization of Cx26 and Cx32 in the rat parotid gland [7] and by the similar cellular localization for Cx26, Cx32, and Cx43, which we observed in human minor salivary glands. However, other studies will be necessary to verify this possible colocalization. In addition, future studies could be performed to verify our very preliminary results on the modulation of the Cx expression in pSS samples and their possible involvement in salivary gland dysfunctions. Finally, this paper aims to be a pilot study that can enable research on the pathogenic mechanisms underlying different salivary gland diseases.

## Figures and Tables

**Table 1:** Details of gene specific primers used in real-time PCR experiments.

Gene	Primer Sequence	GenBank Accession Number	Amplicon Length	Ta, °C
Cx26	Hs_GJB2_1_SG QuantiTect Primer Assay (QIAGEN, Hilden, Germany)—Blind sequence	NM_004004	--------------	60
Cx43	F: CTCAACAACCTGGCTGCGAAA R: GGTGGGCACAGACACGAATAT	NM_012567.2	60 bp	60
Cx32	F: TTGCCTTGCTGCCTGCTA R: ACTCTGATTTATCTGCCTGCTTCT	NM_017251	86 bp	60
ACTB	F: GTCGTACCACTGGCATTGTG R: CTCTCAGCTGTGGTGGTGAA	NM_031144	181 bp	60
RPL13a	F: CGCCCTACGACAAGAAAAAG R: CCGTAGCCTCATGAGCTGTT	NM_012423	206	60
RPS4X	F: GATCCCCTCATCAAGGTGAA R: GCCCTTGCCAATAACAAAAA	NM_002046	243	60

## Data Availability

Data available upon request.

## Supplementary Materials

The following supporting information can be downloaded at: https://www.mdpi.com/article/10.3390/molecules27185926/s1, Figure S1. Representative images of negative controls for immunohistochemistry. (A,C) Confocal microscopy images showing gland tissue by transmitted light contrast and nuclei of cells blue-stained by dapi; (B,D–F) transmission electron microscopy images. Negative controls were obtained: by omitting primary antibodies, as shown in A for IF and in B for IE; by using an irrelevant primary antibody, as shown in C for IF; by using a non-matching secondary antibody, as illustrated for IE in D for Cx26, in E for Cx32, and in F for Cx43. GC, Golgi complex; M, mitochondrium; MSG, mucous secretory granule; MEC, myoepithelial cell; N, nucleus; RER, rough endoplasmic reticulum. Scale bars A, C, 20 µm; D, 200 nm; B, E, F, 500 nm.
