# Supplementary material for: Connexin Expression in Human Minor Salivary Glands: An Immunohistochemical Microscopy Study
Source: Molecules. 2022 Sep 12;27(18):5926. doi: 10.3390/molecules27185926 (PMC9505306; doi:10.3390/molecules27185926)
Supplement: Supplementary file 1 [file molecules-27-05926-s001.zip › molecules-1865181-supplementary.pdf]

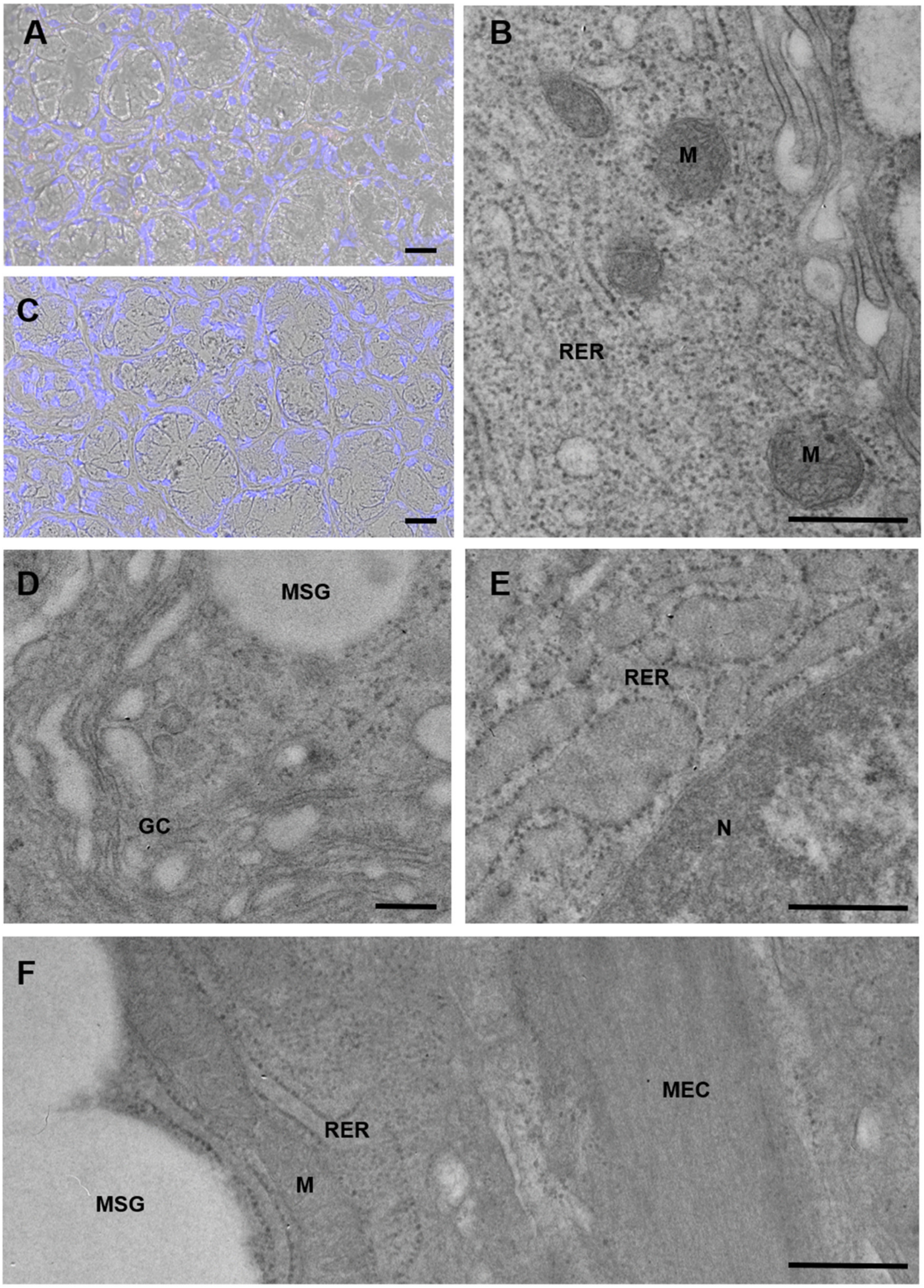


**Figure S1.** Representative images of negative controls for immunohistochemistry. (**A**,**C**) Confocal microscopy images showing gland tissue by transmitted light contrast and nuclei of cells blue stained by dapi; (**B**,**D**-**F**) transmission electron microscopy images. Negative controls were obtained: by omitting primary antibodies as shown in A for IF and in B for IE; by using an irrelevant primary antibody as shown in C for IF; by using non-matching secondary antibody as illustrated for IE in D for Cx26, in E for Cx32 and in F for Cx43. GC, Golgi complex; M, mitochondrium; MSG, mucous secretory granule; MEC, myoepithelial cell; N, nucleus; RER, rough endoplasmic reticulum. Scale bars A, C, 20 µm; D, 200 nm; B, E, F, 500 nm.
